# New vision on the mental problems of Vincent van Gogh; results from a bottom-up approach using (semi-)structured diagnostic interviews

**DOI:** 10.1186/s40345-020-00196-z

**Published:** 2020-11-02

**Authors:** Willem A. Nolen, Erwin van Meekeren, Piet Voskuil, Willem van Tilburg

**Affiliations:** 1 Department of Psychiatry, University Medical Center Groningen, University of Groningen, Hanzeplein 1, 9713 GZ Groningen, The Netherlands; 2 BuurtzorgT, Oeverwallaan 130, 2498 BK The Hague, The Netherlands; 3 Torendreef 14, 4851 BH Ulvenhout, The Netherlands; 4 Department of Psychiatry, Amsterdam University Medical Centers, Vrije Universiteit, De Boelelaan 1117, 1081 HV Amsterdam, The Netherlands

**Keywords:** Vincent van Gogh, Bipolar disorder, Psychotic depression, Borderline personality disorder, Alcohol use disorder, Delirium, Focal epilepsy

## Abstract

**Background:**

On July 29, 1890 at the age of 37 years, the Dutch painter Vincent van Gogh died from the consequences of a suicide attempt with a gun 2 days earlier. Since then many medical and psychological theories were suggested about what had happened to Van Gogh.

**Aim:**

To present an overview of the history of the mental problems of Van Gogh and the most likely diagnoses.

**Method:**

(Semi-)structured diagnostic interviews were applied to three art historians who are very familiar with Van Gogh from his correspondence and other sources as well as a neuropsychiatric examination to evaluate whether the symptoms might be explained by a medical condition.

**Results:**

Several previously suggested diagnoses could be excluded as being highly unlikely, while other diagnoses could be classified as more of less likely.

**Conclusion:**

Most likely Van Gogh suffered from comorbid illnesses. Since young adulthood, he likely developed a (probably bipolar) mood disorder in combination with (traits of) a borderline personality disorder as underlying vulnerability. This likely worsened through an alcohol use disorder combined with malnutrition, which then led, in combination with rising psychosocial tensions, to a crisis in which he cut off his ear. Thereafter, he likely developed two deliriums probably related to alcohol withdrawal, followed by a worsening with severe depressive episodes (of which at least one with psychotic features) from which he did not fully recover, finally leading to his suicide. As additional comorbidity, focal (temporal lobe) epilepsy cannot be excluded.

## Background

On July 29, 1890 at the age of 37 years, the Dutch painter Vincent van Gogh died from the consequences of a suicide attempt with a gun 2 days earlier. Suicidal behaviour usually is the result of a history with mental problems. Although in Van Gogh’s case the beginning of this history is not easy to determine, a critical period in his life started when he cut off his left ear followed by three consecutive hospitalizations in Arles between December 1888 and May 1889, and subsequently a transfer to the asylum of Saint-Rémy-de-Provence in May 1889, where he arrived having “absolutely no will, hardly any desires or none” [776].^1^ He stayed in Saint-Rémy for a full year but did not fully recover, and also a move to Auvers-sur-Oise did not bring the relief he wished. That his existence at that time appeared “attacked at the very root” [898] was possibly the reason for his suicide.

Van Gogh himself initially did not understand what was wrong with him. He wrote about a “mental or nervous fever or madness, I do not know quite what to say or how to name it” [739], and initially—possibly to reassure himself, his friends and family—also of “a simple artist’s bout of craziness” [732]. However, Van Gogh’s own doctors came to various diagnoses. Felix Rey, his doctor in Arles, called his first crisis when he cut off his ear “a transient over-excitement” [728] and blamed it on his lifestyle with much alcohol, coffee, tobacco and poor food, while Rey’s chief Jules Urpar noticed “an attack of acute mania with generalized delirium” [772, note 2]. When the episodes returned Rey further suggested epilepsy as the cause [776], which was confirmed by Théophile Peyron, his doctor in Saint-Rémy: “attacks of epilepsy, separated by long intervals” [772, note 2]. Subsequently, after his death a comprehensive discussion arose not only on the above but also on many other suggested diagnoses and theories about what had happened to Van Gogh (Bakker et al. 2016). And finally, Van Gogh’s extensive correspondence further inspired the discussion.

In this paper we will try to contribute to a better understanding of the psychopathology of Vincent van Gogh. Many of the previously suggested diagnoses resulted from a ‘top-down approach’, i.e. were based on the assumption that Van Gogh suffered from a specific illness based on arguments pro that illness in his letters and other sources without taking into account other information making that illness unlikely. In contrast, we used a ‘bottom-up approach’ by applying (semi-)structured interviews to assess all mental symptoms ever reported by Van Gogh in his letters or as found in other sources. Thus, we aimed to explore all possible diagnoses, in order to exclude (very) unlikely illnesses and leaving other illnesses as more or less likely in the case of Van Gogh.

## Methods

### General considerations

For our study we used several diagnostic approaches. However, before applying these, we had to realize whether it would be allowed to make a diagnosis without having Van Gogh interviewed and examined in person. In fact, the medical code of ethics is generally very cautious about this point (APA Calls for End to ‘Armchair’ Psychiatry 2020). Nevertheless, in this case we think that we can not only exclude several of the previously suggested diagnoses with certainty, but also be more or less sure about other parts of the diagnostic process. This is based on the fact that we have access to his extensive correspondence containing 902 letters, of which 820 were written by him to his brother Theo and other relatives. In these letters he described what he was experiencing in his life, including his mental problems, although it must also be realized that Van Gogh did not write his letters for his doctors, but for his brother Theo and other relatives, to inform, or to reassure them.

### Diagnostic assessments

In a first step we profited from the knowledge of three art historians very familiar with Van Gogh as a person, from his correspondence as well as from several other sources. To ‘use this knowledge’ we interviewed them with three diagnostic assessments. The first was the ‘Structured Clinical Interview for DSM-IV’ (SCID-IV)^2^ applied by one of the authors (WAN) (First et al. 2002). The SCID scoring system was slightly modified: any possible symptom ever reported in his life in the sources was scored as ‘certainly not’, ‘possibly’, ‘probably’ or ‘certainly’ present or as ‘unclear’. In a next step we searched the whole correspondence for citations that could confirm the responses of the art historians to the SCID-IV. We present the most clear citations in the historical overview of Van Gogh’s mental problems.

The same art historians were interviewed by another author (EVM) using four questionnaires on personality disorders [the ‘Self Test Viersprong’ (https://www.deviersprong.nl/wp-content/uploads/); the ‘McLean Screening Instrument for Borderline Personality Disorder’ (MSI-BPD) (Zanarini et al. 2003); the ‘General Assessment of Personality Disorders’ (GAPD) (Hentschel and Livesley 2013); and the ‘Personality Inventory for DSM-5’ (PID 5)] (Thimm et al. 2016) about the existence of possible personality pathology while focussing on his life before the ear incident. The answers to these questionnaires were then scored by two independent experienced diagnosticians (T. Ingenhoven, psychiatrist and H. Berghuis, clinical psychologist), who were unfamiliar with the origins of the investigation and the identity of the ‘patient’, i.e. Van Gogh.

Finally, the same art historians were questioned by another author (PV) for a neuropsychiatric examination to evaluate whether the symptoms might be explained by a medical condition, i e. epilepsy.

## Historical overview of the mental problems of Van Gogh

Already from his early adulthood Van Gogh experiences mental problems. His father writes “he always has the inclination to melancholy” [102; note 53]. At about 20 years old (1873–1875, living in London) he is often gloomy, withdraws himself from other people, is focused on religious issues, and—partly because of these behaviours—he loses his job as an art dealer. In the years 1875–1877 (Paris), he shows a strict ascetic attitude, combined with an unhealthy lifestyle. In 1877–1878 (Amsterdam) he writes “My head is sometimes numb and is often burning hot, and my thoughts are confused” [117] and he mentions “indescribably strong feeling of fear” [141]. He also injures himself “Last night I used the cudgel again” (Mendes Da Costa 2020). In 1880 (Borinage) he describes himself as “a man of passions, capable of and liable to do rather foolish things for which I sometimes feel rather sorry. I do often find myself speaking or acting somewhat too quickly when it would be better to wait more patiently” [155]. He behaves so strange that his father suggests him to go the psychiatric asylum of Geel which Van Gogh refuses [185, 186]. Shortly thereafter he regains his artistic activities. Late 1881 (Etten) he writes several letters which in comparison with the rest of his correspondence, are remarkably long and also rather incoherent [180–190].

In 1882–1883 (The Hague) he feels “as though one is bound hand and foot, lying in a deep, dark pit, powerless to do anything” [203] and reports fear and sorrow which “cannot but make one agitated and nervous in speech and manner” [221]. Moreover, he mentions that he doesn’t enjoy company, and that dealing with people, talking to them, is often painful and difficult [244]. Nevertheless, he remains active with drawing and painting.

Late 1883 (Drenthe) he “is overcome by a feeling of great anxiety, dejection and even despair, too much to express”. Next (Nuenen) he senses a “procrastination and hesitancy in everything, which paralyzes my own passion and energy like a leaden atmosphere” [410]. A few months later (1884) he is working “from early till late” and being “absorbed in the moment”, in a letter also containing 10 poems [430]. He remains very active for several months, but finally he writes “I’ve worked really hard recently; I believe, in conjunction with other agitation, even overworked. At least I’m in a sad mood, and all these things have affected me to such an extent that I have many days when I’m relatively powerless. I can’t eat and I can’t sleep, that’s to say not enough, and that makes one weak” [463]. In this period he leaves the church and becomes engaged in inappropriate love affairs which also leads to a further disturbed relationship with his parents.

In 1885 Van Gogh moves to Antwerp and after a few months to Paris (1886–1887), where he goes to live with Theo, which means that he temporarily stops writing letters. In Paris, he has “the most impossible and highly unsuitable love affairs from which, as a rule, I emerge only with shame and disgrace” [574] combined with (probably) substantial alcohol use and heavy smoking [603].

In 1888 Van Gogh moves to Arles where initially he is very active “in a fury of work” [592] and experiences a “rage to paint orchards [which] won’t last forever” [594]. However, a month later he reports “ It’s not in black that I see the future, but I see it bristling with many difficulties, and at times I wonder if these won’t be stronger than I am”, and “I was so worn out and ill that I didn’t feel I had the strength to go and live on my own” [602, 608]. He continues drinking and smoking “the only thing that comforts and distracts—in my case—as in others, is to stun oneself by taking a stiff drink or smoking very heavily” [635]; also to find relief “If the storm within roars too loudly, I drink a glass too many to stun myself. It’s being crazy, compared with what one ought to be” [645]. He has days being active with painting, but also days feeling “thoroughly discouraged” and “My life is restless and anxious” [672]. Furthermore, he reports a changed sleeping pattern: “For 3 nights I stayed up to paint, going to bed during the day. It often seems to me that the night is much more alive and richly coloured than the day” [676]. Additionally, he realizes the alternating pattern of his mental problems: “I have a terrible clarity of mind at times, when nature is so lovely these days, and then I’m no longer aware of myself and the painting comes to me as if in a dream. I am indeed somewhat fearful that that will have its reaction in melancholy when the bad season comes” [687].

In October 1888 Gauguin joins Van Gogh in Arles. Initially, their collaboration is fruitful, but soon tensions arise. Already after 6 weeks Gauguin expresses the idea to leave as he “can absolutely not live side by side without trouble, as a result of incompatibility of temperament, and both he and I need tranquillity for our work” [724, note 1]. In the night of December 22 Gauguin decides to leave. This leads to a crisis in which Van Gogh directs the aggression towards himself. On the evening of December 23, he cuts off his left ear and gives it away to a woman in a brothel.

Alarmed police finds him the next morning at home suffering from blood loss, and he is admitted to the local hospital. Two days later (December 26) he becomes so confused that he is put in isolation: “My thoughts sailed over many seas. I even dreamed of the Dutch ghost ship and the Horla, and it seems that I sang then” [739]. Afterwards he has only vague memories. He also has vivid visual images: “I again saw each room in the house at Zundert, each path, each plant in the garden, the views round about, the fields, the neighbours, the cemetery, the church, our kitchen garden behind—right up to the magpies’ nest in a tall acacia in the cemetery” [741] and also “unbearable hallucinations”, anxiety and nightmares [743]. Another 7 days later (January 2, 1889), he is recovered and discharged from the hospital on January 7. At that time, he thinks not much is wrong, but afterwards he has the feeling that he has been ill: “What can you say, I have moments when I’m twisted by enthusiasm or madness or prophecy like a Greek oracle on her tripod” [745]. He resumes his activities but within a month it goes wrong again.

On February 7, he is re-admitted with apparently similar symptoms and again placed in isolation. In a report, on request of the Chief of Police, doctor Albert Delon writes “I found this man in a state of extreme excitement suffering from a true delirium, only momentarily recognizing the people around him”, and he hears hallucinations [747, note 1]. It is unclear whether he was drinking alcohol again between his first and second admission. Again his hospital stay is short, and on February 17 he goes back to work. However, there are complaints from a group of 30 residents about nuisance and inappropriate behaviour, e.g. touching women and making obscene remarks (The Arles Petition 1889). This results in the third and this time involuntary admission on February 27. Whether he is confused or just angry by the complaints about his behaviour and that he is placed in isolation, remains unclear [750]. Afterwards, he also mentions “terrible fits of anxiety sometimes without any apparent cause” [764].

**Fig. 1:**
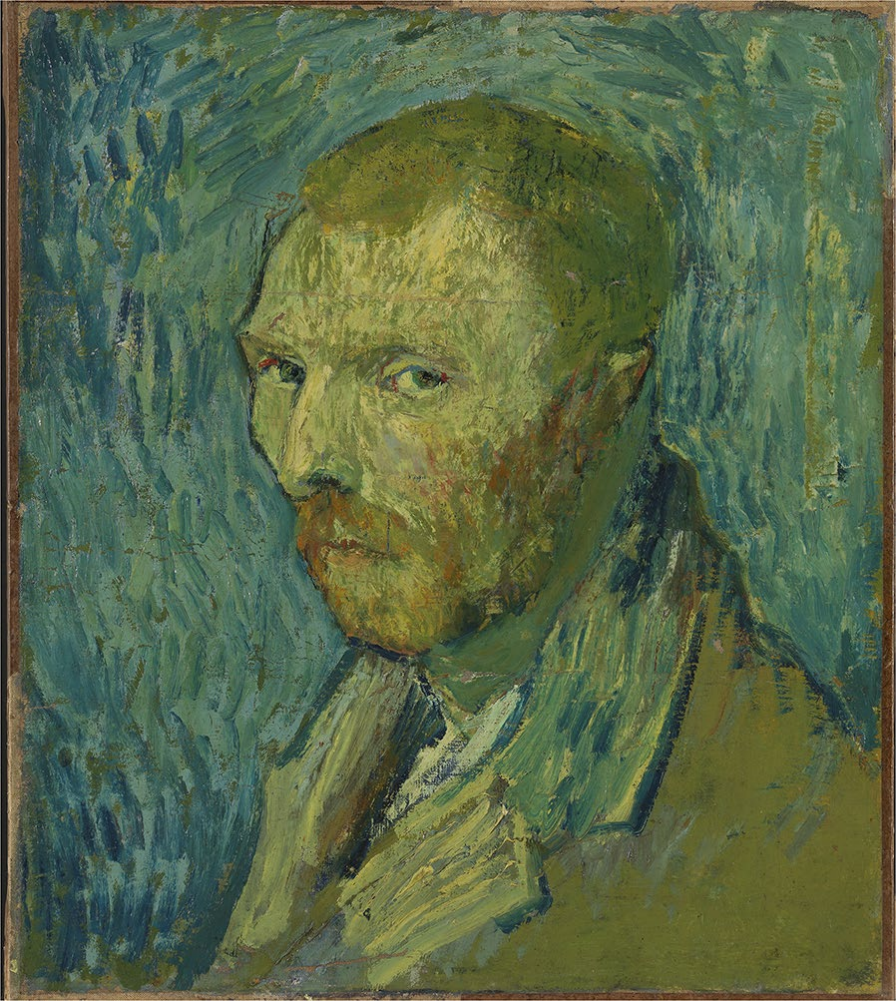
Self-portrait (August 1890), Nasjonalmuseet, Oslo, Norway

Mid-March he has almost recovered and goes back to home although he continues to eat and sleep in the hospital while feeling uncertain about the future: “It will, I hope, suffice to say that I feel decidedly incapable of starting to take a new studio again and living there alone”. In addition, he expresses concerns about his alcohol use [760]. Looking back to what happened in the previous months, he mentions four (?) “big crises in which I hadn’t the slightest idea of what I said, wanted, did”, while he can’t precisely describe what it is like: “there are terrible fits of anxiety sometimes—without any apparent cause—or then again a feeling of emptiness and fatigue in the mind. I consider the whole rather as a simple accident, no doubt a large part of it is my fault, and from time to time I have fits of melancholy and terrible feelings of guilt” [764]. He agrees with the suggestion of his doctor and Theo to agree to a voluntary admission in the asylum in Saint-Rémy on May 8, 1889 where he will stay for a year, experiencing four episodes/crises. The first episode lasts longer (from mid-July to late August 1889) than the previous three crises in Arles. He complains about a “disturbed mind” and is “absolutely distraught, as in Arles, just as much if not worse”. [797] He also shows bizarre behaviour: “It appears that I grab dirt from the ground and eat it, although my memories of these bad moments are vague” [797]. When looking back to this period he writes: “I feel cowardly in the face of anguish and suffering—more cowardly than is justified”, recognizing that the crises “tend to take an absurd religious turn” and mentioning “I reproach myself for my cowardice” [801]. He also he paints himself in a recently to Van Gogh attributed self-portrait (Fig. 1) (Van Tilborgh et al. 2020).

Late December 1889 follows a short crisis lasting 1 week with “great discouragement” [836] while his guard Poulet observes that he swallows paint what is interpreted by his doctor Peyron as an attempt to poison himself [833, note 2]. On January 21, 1890, 2 days after a visit to Arles, he is again seized by an attack lasting about a week. According to his doctor Peyron “He is incapable at present of any kind of work, and only responds with incoherent words when asked questions” [846, note 2]. A few days after having received a letter from Theo announcing that he has become a father, Van Gogh congratulates him: “It does me more good and gives me more pleasure” and it “contributes a great deal to making me forget these last few days when I was ill, then I no longer know where I am and my mind wanders” [850].

However, a prolonged and severe episode again follows from February to April 1890, where he is “totally dazed”. [857], “not able to read or write” and “ill at its worst” “[864]. Disappointed that he has not recovered in Saint-Rémy he then takes the decision to leave for the North [863, 865]. In May 1890 he moves to the rural Auvers-sur-Oise near Paris. However, he was again unable to make a lasting recovery and finally he shoots himself in the breast on July 27 1890, ending his life 2 days later (Van Tilborgh and Meedendorp 2013).

## Results

In the following paragraphs we present of the findings of the diagnostic assessments and our interpretations. For more extensive information see Additional file 1: Tables S1–S11.

### Psychotic disorder?

It is certain that Van Gogh suffered from at least three episodes with psychotic symptoms (Additional file 1: Table S3). The first two, in December 1888 (after the ear incident) and in February 1889, were short (lasting only a few days) and consisted of vivid hallucinations, with afterwards partial amnesia (indicating a decrease of consciousness) and cognitive dysfunction. According to the SCID-IV these two psychotic episodes appear to have been a delirium. The third episode occurred in July–August 1889 in Saint-Rémy, when there were apparent religious delusions, but this time no hallucinations. He was also severely depressed with self-reproach, his thinking was possibly delayed, he exhibited bizarre behaviour (swallowing paint) and his memory of it was vague. The neurologist/epileptologist among the authors (PV) assumes that this memory disturbance indicates a decrease of consciousness, while the psychiatrists (WN, EVM and WVT) are of the opinion that is can be explained by a severe depression with psychotic features. Regarding the other three crises in Saint-Rémy it is unclear whether he was psychotic and/or had a decreased consciousness.

After Van Gogh’s death one of the first suggested diagnoses (1922) was schizophrenia (Jaspers 1922). However, with the current understanding of this disorder, this seems unlikely as van Gogh never exhibited psychotic symptoms before the ear incident at the age of 35, and also not during the intervals between his psychotic episodes in the last 15 months of his life. Moreover, there is no indication that he suffered from negative symptoms such as diminished emotional expression or avolition. This makes the diagnosis schizophrenia highly unlikely.

Another suggestion was cycloid psychosis, characterized by confusion states, psychotic symptoms, mood swings, anxiety and psychomotor disturbances (Leonhard 1988). Nowadays, cycloid psychosis is defined by the occurrence of recurrent short psychotic episodes with delusions, hallucinations, incoherence or disorganized behaviour which cannot be explained by a major mood disorder, schizophrenia, a general medical condition or associated substance abuse (Fusar-Poli et al. 2016). Although this diagnosis can be considered in Van Gogh’s case, it has common features with other explanations, like a mood disorder or a personality disorder. In our opinion, these alternative explanations make cycloid psychosis as primary diagnosis unlikely.

### Unhealthy lifestyle?

**Fig. 2:**
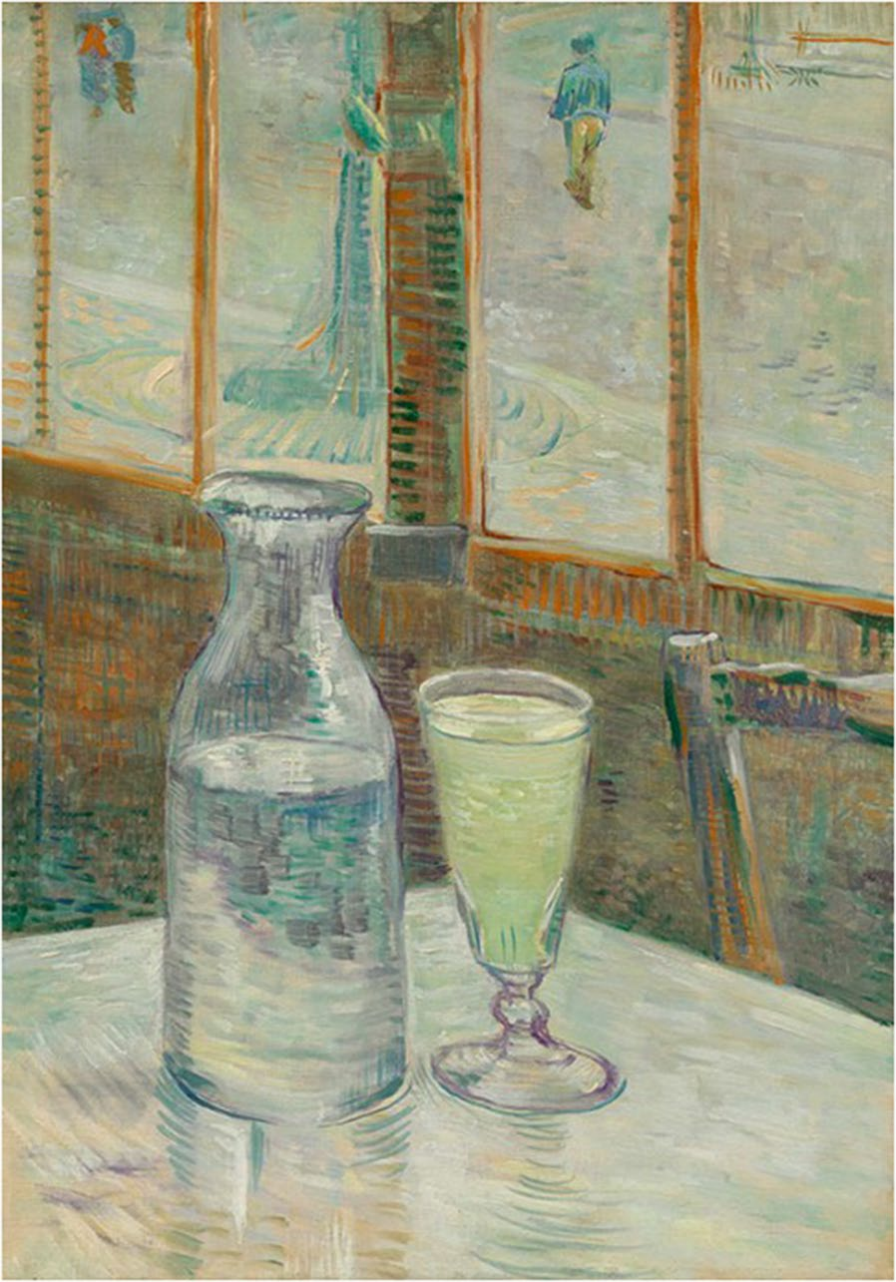
Still life: Café table with absinthe (February/March 1887), Van Gogh Museum, Amsterdam, The Netherlands

Definitely since 1886, but possibly earlier, Van Gogh drank very much alcohol, and he tried to reduce it in Arles, but without success, indicating that he was dependent on alcohol. Besides wine, he also drank absinthe which at that time contained 50 to 70% alcohol (Fig. 2). In addition, there are many indications of malnutrition and poor sleeping habits, if not exhaustion. Those who consume large amounts of alcohol in combination with malnutrition, run the risk of brain function impairment including mental problems. Moreover, abrupt stopping with excessive alcohol consumption can lead to withdrawal phenomena, including a delirium. Therefore, it is likely that at least the first brief psychosis in Arles on the days after the ear incident during which he likely stopped drinking abruptly, was actually an alcohol withdrawal delirium. Only later on in Saint-Rémy, when he was forced to minimize or even stop drinking, he probably succeeded in it and he also did not have further withdrawal problems.

### Mood disorder?

In many letters Van Gogh reported depressive symptoms (Additional file 1: Table S2). From 1874 (London) up to the ear incident (1988), he almost certainly suffered from several depressive episodes. Moreover, in Saint-Rémy he had at least two major depressive episodes (July–August 1889 and February-April 1890), with also psychotic features in the first episode (discussed above), and with severe distress and disfunction in both episodes. He also reported manic symptoms, possibly during his stay in London, very likely in the Borinage, and in his 1st year in Arles. There were also periods with a decreased need for sleep and high, increased activity; whether he also showed social disfunction in these periods is not fully clear.

Since the 1930s it has been suggested that van Gogh suffered from bipolar disorder (Rose and Mannheim 1938; Jamison 1993). Although Van Gogh showed mood swings and according to his brother Theo was as if he consisted of two conflicting personalities “the one marvellously gifted, sensitive and gentle, and the other self-loving and unfeeling” [FR b908], the question remains whether the diagnostic threshold of a manic episode with social disfunction has ever been exceeded at some point in its life, meaning that his episodes with manic symptoms were only hypomanic episodes. Thus, it is likely that van Gogh suffered from a bipolar disorder, and if not bipolar 1 disorder then probably bipolar 2 disorder. Nevertheless, it must also be considered that his mood symptoms were part of a personality disorder.

### Personality disorder?

Van Gogh was unstable in his personal relationships, starting with his father and from then on with many other people in the different places where he lived. Even with his so beloved brother Theo there sometimes were tensions, especially during their time living together in Paris (1886) and on the subject of how he should behave towards their father. Early on he exhibited solitary behaviour, while he also had a great need to meet the Christian duties of charity. He idealized family life, but he failed to marry and to found a family himself despite he had several, although problematic, love affairs. Living with family or artists was the only alternative, but in both respects he was no ideal partner. As from 1880 (Borinage) he showed self-neglect and from 1886 (Paris) also self-destructive behaviour (drinking too much alcohol combined with malnutrition), self-mutilation [the ear incident (1888) and possible self-poisoning (1889)] and ultimately (1890) he committed a suicide attempt that resulted in his death. This raises the question whether these mental problems were part of a personality disorder and more specific a borderline personality disorder—as suggested by several authors since 1996 (Mehlum 1996; Van Meekeren 2000; Van Meekeren 2004)—with a persistent pattern of instability of interpersonal relationships, the self-image and emotions, and marked impulsivity, often combined with self-destructive behaviour such as suicidal behaviour or self-mutilation.

The conclusions of the consulted diagnosticians who scored the four personality disorder questionnaires, were ‘clear suggestions for a personality disorder’ (Additional file 1: Table S6–S8). All questionnaires contain strong indications for a personality disorder, while the combination of the different traits convincingly indicate a severe borderline personality disorder, given the score of 8 on the MSI-BPD and that all DSM-5 criteria are fulfilled. There are also traits of an obsessive–compulsive personality disorder, however not in the compulsive, controlling sense but in the sense of a perseverative, rigid perfectionism.

### Somatic disorder?

Over the years, several somatic diagnoses have been suggested, such as Meniere’s disease (Arenberg et al. 1990; Martin 2011), (neuro)syphilis (the disease that Theo died from a half year after Vincent) (Yamey 2003), acute intermittent porphyria (AIP) exacerbated by drinking absinthe containing thujone (Loftus and Arnold 1991), and various forms of intoxications including carbon monoxide poisoning.

All these suggestions are considered highly unlikely. The sources give no reason to think that he was suffering from Meniere’s disease. While syphilis occurred frequently in Van Gogh’s time and was well known, none of his doctors made this diagnosis, even not after they treated him for gonorrhoea. As an extremely rare autosomal genetic condition AIP is unlikely as none (even later) family members is known with this disease. In addition, thujone intoxication was rejected, first by the unlikeness that he suffered from AIP, and second by the fact that absinthe at Van Gogh’s time contained a very low concentration of thujone (Bonkovsky et al. 1992). Carbon monoxide poisoning due to gas lamps in his home in Arles is also unlikely as there are no other reports of (possible) carbon monoxide poisoning in Arles (Van Slooten 2020).

### Epilepsy?

The outcome of the neuropsychiatric interview is discussed in Additional file 1: Table S9–S11. When Van Gogh’s doctors concluded that he had epilepsy, they probably meant ‘épilepsie larvée mental’ (Morel 1860). This form of epilepsy is also called ‘masked epilepsy’, wherein a patient does not have classical seizures, but a paroxysmal behaviour disorder based on epileptic activity in the deeper brain structures. This diagnosis is nowadays called temporal lobe epilepsy or recently focal epilepsy with focal onset seizures, resulting in a highly variable expression of anxiety, delusions and hallucinations, depending on the affected part of the mesial temporal network (Blumer 2002).

A possible explanation of such encephalopathic aetiology from the end of 1888 is then found in `Van Gogh’s lifestyle with alcohol abuse, malnutrition, poor sleep and mental exhaustion. However, without further examination by electroencephalographic and imaging techniques (not available in his time) the probability of epilepsy is difficult to quantify. Therefore, focal (temporal lobe) epilepsy as comorbidity to the above discussed psychotic disorders, mood disorders and personality disorders, cannot be excluded and deserves at least to be considered (Additional file 1: Table S11) (Voskuil 2013; Voskuil 2020).

## Discussion and conclusions

As far as we know this study is the first in which (semi-)structured diagnostic interviews assessing all possible mental symptoms, were applied in a historic person to make a psychiatric diagnosis posthumously. We think this ‘bottom-up approach’ is valid in the case of Vincent van Gogh as he has reported so many mental symptoms in his extensive correspondence. Furthermore, our findings are supported by medical reports of the doctors who have treated him. Thus, we consider our findings as rather robust. However, we also want to repeat that Van Gogh did not write his letters for his doctors and that we have not interviewed and examined Van Gogh in person.

Our main conclusion is that in the case of Vincent van Gogh no single disorder can explain all his mental problems throughout his life, but that he most likely suffered from several comorbid disorders. Starting at young adulthood (i.e. long before the ear incident in December 1988), he frequently reported mental problems and had difficulties in relating with people. He also was regularly depressed, had probably periods with manic symptoms, drank much alcohol and did not take enough care of himself at several occasions. Overlooking this period, it seems appropriate to conclude that Van Gogh showed—in today’s terminology—identity and attachment problems and/or personality problems, more specifically with borderline features. Depending on the evaluation whether or not there was an enduring pattern with impairment, he had a personality disorder or traits thereof. In addition, there appeared to be an evolving mood disorder, more particularly recurrent depressive episodes probably in the course of a bipolar disorder.

**Fig. 3:**
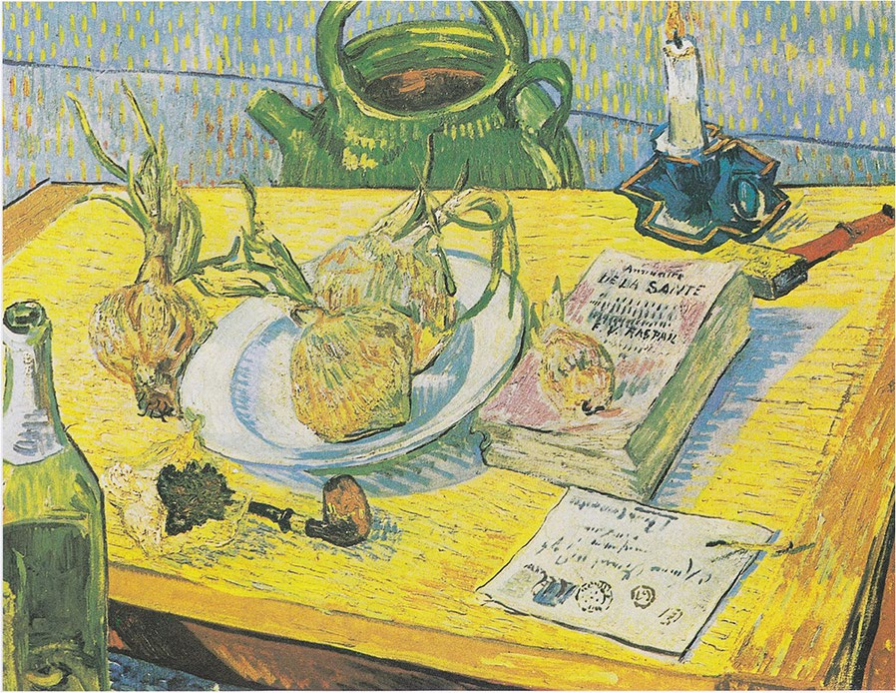
Still life: Drawing board, pipe, onions and sealing-wax (January 1889), Kröller-Müller Museum, Otterloo, The Netherlands

As of the end of 1888, his mental and somatic health deteriorated, preceded by an increase of alcohol consumption since 1986 combined with malnutrition (Fig. 3), resulting in the ear incident. Subsequently, there were three successive crises. In two of these crises (December 1888–February 1889) he showed features of a delirium, possibly after abrupt stopping with alcohol. After his move to Saint-Rémy (May 1889), his mood disorder got worse with several severe depressive episodes of which the first one (July–August 1889) most likely was a psychotic depressive episode. Another theoretical explanation of (part of) the symptomatology after December 1888 is focal (temporal lobe) epilepsy, either as co-morbidity or as an alternative—i.e. differential—diagnosis.

Especially the worsening of his alcohol use since 1886 appears relevant, as this seems to be part of the reason—besides conflicting strong characters with different opinions on life and art—why Gauguin decided to leave Arles as well as why Van Gogh could develop a delirium after he abruptly stopped drinking. Moreover, it can theoretically explain the development of focal (temporal lobe) epilepsy. In addition, there were several other psychosocial factors that played a role: concerns about the continuation of the financial support by Theo and maybe also about his brother’s health, the imminent failure of creating a family or instead a brotherhood of painters, and finally his lack of success as a painter (he sold just one painting).

**Fig. 4:**
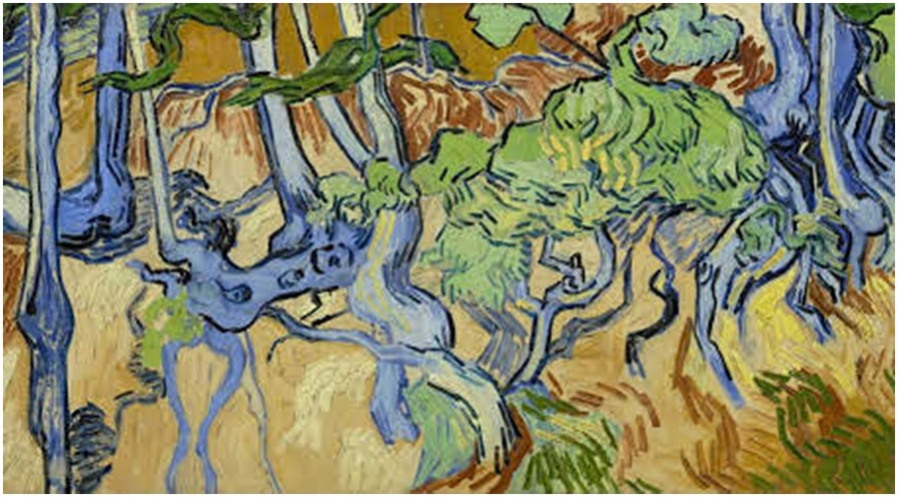
Tree roots (July 1890), Van Gogh Museum, Amsterdam, The Netherlands

Despite all these problems which contributed to his illnesses, we however also would like to stress that Van Gogh was not only a great and very influential painter but also an intelligent man with an enormous willpower, resilience and perseverance. He must have had a strong constitution. He was able to arouse compassion, himself having compassion with the less fortunate. And he was a passionate man with a strong temperament. Over the years he kept on painting, also during most difficult periods in his life. Only during the most severe psychotic episodes he temporarily stopped working, but in intervals with less symptoms he was able to paint. Only in a few paintings a relationship with his mental state is evident, e.g. in the ‘Oslo self-portrait’ (Fig. 1) painted at the end of his psychotic depressed episode in July–August 1889 (Van Tilborgh et al. 2020). Regarding the unfinished ‘Tree roots’ (Fig. 4) from July 1890, Van Gogh’s last work and painted on the morning before he shot himself, the question is whether he depicted there his struggle for life. Art historians think it is unlikely, “food for biographers and another story” (Maes and Van Tilborgh 2012).

## Supplementary information

**Additional file 1: Table S1.** Overview of scores on relevant items of the SCID-IV: Screening module. **Table S2.** Overview of scores on relevant items of the SCID-IV: Module mood disorders. **Table S3.** Overview of scores on relevant items of the SCID-IV: Module psychotic disorders. **Table S4.** Overview of scores on relevant items of the SCID-IV: Module anxiety disorders. **Table S5.** Overview of scores on relevant items of the SCID-IV: Module substance use disorders. **Table S6.** Conclusions from questionnaires on personality disorders. **Table S7.** Scores on the General Assessment of Personality Disorders (GAPD). **Table S8.** Scores on the individual items of the Personality Inventory for DSM-5 (PID-5). **Table S9.** Neuropsychiatric history of Van Gogh. **Table S10.** Neuropsychiatric family history of Van Gogh. **Table S11.** Arguments pro and contra epilepsy as a possible diagnosis in Van Gogh’s case.

## Data Availability

Not applicable.

## Footnotes

1 The numbers between brackets refer to the letters in Van Gogh’s correspondence: Jansen L, Luijten H, Bakker N (eds.), Vincent van Gogh. The Letters, The Hague 2009, yearly updated, http://vangoghletters.org/vg/.

2 At the time of the interview the SCID-5 was not yet translated into Dutch.
