# Correction: New vision on the mental problems of Vincent van Gogh; results from a bottom-up approach using (semi-)structured diagnostic interviews

**DOI:** 10.1186/s40345-022-00273-5

**Published:** 2022-11-14

**Authors:** Willem A. Nolen, Erwin van Meekeren, Piet Voskuil, Willem van Tilburg

**Affiliations:** 1 Department of Psychiatry, University Medical Center Groningen, University of Groningen, Hanzeplein 1, 9713 GZ Groningen, The Netherlands; 2 BuurtzorgT, Oeverwallaan 130, 2498 BK The Hague, The Netherlands; 3 Torendreef 14, 4851 BH Ulvenhout, The Netherlands; 4 Department of Psychiatry, Amsterdam University Medical Centers, Vrije Universiteit, De Boelelaan 1117, 1081 HV Amsterdam, The Netherlands

## Correction: Int J Bipolar Disord (2020) 8:30 https://doi.org/10.1186/s40345-020-00196-z

In this article, the sentence “From 1874 (London) up to the ear incident (1988), he almost certainly suffered from several depressive episodes”, the year “1988” should be “1888” and in the sentence “As of the end of 1888, his mental and somatic health deteriorated, preceded by an increase of alcohol consumption since 1986 combined with malnutrition”, the year “1986” should be “1886”.
