# Supplementary material for: New vision on the mental problems of Vincent van Gogh; results from a bottom-up approach using (semi-)structured diagnostic interviews
Source: Int J Bipolar Disord. 2020 Nov 2;8:30. doi: 10.1186/s40345-020-00196-z (PMC7604278; doi:10.1186/s40345-020-00196-z)
Supplement: Supplementary file 1 — Additional file 1: Table S1. Overview of scores on relevant items of the SCID-IV: Screening module. Table S2. Overview of scores on relevant items of the SCID-IV: Module mood disorders. Table S3. Overview of scores on relevant items of the SCID-IV: Module psychotic disorders. Table S4. Overview of scores on relevant items of the SCID-IV: Module anxiety disorders. Table S5. Overview of scores on relevant items of the SCID-IV: Module substance use disorders. Table S6. Conclusions from questionnaires on personality disorders. Table S7. Scores on the General Assessment of Personality Disorders (GAPD). Table S8. Scores on the individual items of the Personality Inventory for DSM-5 (PID-5). Table S9. Neuropsychiatric history of Van Gogh. Table S10. Neuropsychiatric family history of Van Gogh. Table S11. Arguments pro and contra epilepsy as a possible diagnosis in Van Gogh’s case. [file 40345_2020_196_MOESM1_ESM.pdf]

Supplemental Tables

Supplemental Table 1. Overview of scores on relevant items of the SCID-IV: Screening module

Nr.	**SCREENING QUESTIONS**	**?**	**No**	**Ps**	**Pr**	**Yes**	**Source** [1]
P1	Ever 5 or more drinks/occasion?					X	
P2	Ever used street drugs?		X				
P3	Ever hooked on prescribed medicine or taken lot more?		X				
P4	Ever had a panic attack?			X			779
P5	Ever afraid of going out the house alone?			X			
P6	Ever felt afraid or uncomfortable doing things in front of people?	X					
P7	Ever nervous/anxious in social situations with unknown people?				X		
P8	Ever afraid of special things/situations?		X				
P9	Ever bothered by senseless thoughts that are coming again?		X				
P10	Ever doing things over and over again?		X				

No = certainly not present; Ps = possibly present; Pr = probably present; Yes = certainly present

[1] The numbers refer to the letters in Van Gogh's correspondence

Supplemental Table 2. Overview of scores on relevant items of the SCID-IV: Module mood disorders

Nr.	**DEPRESSIVE EPISODE**	?	No	Ps	Pr	Yes	Source [1]
A1	Depressed mood				X		221, 230, 241, 244, 390, 463, 672, 764, 815, 836, 874, 856
A2	Diminished interest/pleasure					X	117, 141, 410, 776, 779, 804
A4	Significant weight loss / decreased appetite					X	463
A5	Significant weight gain / increased appetite	?					
A7	Insomnia					X	230, 410, 463, 735
A8	Hypersomnia			X			704
A10	Psychomotor retardation			X			611
A11	Psychomotor agitation	X					
A10	Fatigue/loss of energy					X	117, 410, 611, 764
A11	Worthlessness or guilt					X	117, 764, 801, 831
A12	Diminished thinking/concentration				X		611, 779, 815, 857, 864
A13	Recurrent thoughts or death/suicide(-attempt)					X	797, 833, July 27 1890
	A1-A13: ≥5, incl. A1 or A2					X	
A25	Significant distress/impairment					X	221, 230, 608, 864
A15	Not substance related of medical				X		
A16	Not bereavement					X	
	**Conclusion:**<br>Depressive episodes					X	
Nr.	**(HYPO)MANIC EPISODE**	?	No	Ps	Pr	Yes	Source
A82	Elevated, expansive or irritable mood (≥1 week or ≥4 days)					X	592, 180-190 (all)
A87	Inflated self-esteem/grandiosity			X			709
A88	Decreased need for sleep					X	676
A89	More talkative	X					
A90	Flight of ideas				X		180-190 (all)
A91	Distractibility	X					
A92	Increased goal-directed activity					X	155, 430, 592, 594, 666, 676, 709
A93	Excessive pleasurable but painful activities			X			574
	A82-A 93: ≥3 incl. A82 = elevated/ expansive or ≥4 incl. A82 = irritable					X	
A97	Marked impairment			X			574, 592, 181-193 (all)
A98	Not substance related of medical						
	**Conclusion:**<br>- Manic episode(s)<br>- Hypomanic episode(s) (if not manic episodes)			X<br>(Manic)		X<br>(Hypomanic)	
	**Overall Conclusion: Differential diagnosis**<br>- Bipolar 1 disorder<br>- Bipolar 2 disorder (if not bipolar I)			X<br>(Bipolar 1)		X<br>(Bipolar 2)	

No = certainly not present; Ps = possibly present; Pr = probably present; Yes = certainly present

[1] The numbers refer to the letters in Van Gogh's correspondence

Supplemental Table 3. Overview of scores on relevant items of the SCID-IV: Module psychotic disorders

Nr.	**PSYCHOTIC SYMPTOMS**	**?**	**No**	**Ps**	**Pr**	**Yes**	**Source** [1]
B1	Delusion of reference		X				
B2	Persecutory delusion			X			747 (note 1)
B3	Grandiose delusion	X					
B4	Somatic delusion	X					
B5	Other delusion<br>- Poisoning delusion<br>- Religious delusion<br>- Guilt delusion			 <br><br>X	 <br>X	 X	 - 747 (note 1)<br>- 801, 805<br>- 764, 801, 831
B16	Auditory hallucination					X	743, 776, Dr Delon
B19	Visual hallucination					X	739, 741, 776, 812
B21	Other hallucination		X				
B24-B28	Catatonic behavior		X				
B29	Disorganized behavior		X				
B30	Inadequate affect		X				
B31	Disorganized speech		X				
B32-B37	Negative symptoms		X				
C22-C24	Schizoaffective criteria		X				
C41-C42	Not associated with medical disorder<br>- porphyria					 X	
	Not associated with with<br>- substance intoxication<br>- alcohol<br>- other (e.g. absynth)<br>- substance withdrawal (e.g. delirium)<br>- alcohol<br>- other	 <br>X	 <br><br><br><br>X			 <br><br>X<br><br><br>X	
Nr.	**DELIRIUM**	?	No	Ps	Pr	Yes	
NA	Disturbance of consciousness				X		797, 812
NA	Change in cognition				X		745, 812, FR b1055, FR b1057
NA	Develops<br>- Within hours/days<br>- And fluctuates over the day	 <br>X				 X	 - 728, FR 1056
NA	Evidence that it developed<br>- During intoxication<br>- During or shortly after withdrawal	 X			 <br>X		
	**Conclusion:**<br>Delirium: all criteria yes				 X		
	**Overall Conclusion: Differential diagnosis**<br>- Psychosis, e.g. schizophrenia or schizoaffective disorder<br>- Depressive episode(s) with psychotic features (July/August 1889)<br>- Deliriums (December 1888-Feburary 1889)		 X		 <br>X<br><br>X		 - 801, 805<br>- FR b1055<br>FR 1056<br>FR b1057

No = certainly not present; Ps = possibly present; Pr = probably present; Yes = certainly present; [1] The numbers refer to the letters in Van Gogh's correspondence

Supplemental Table 4. Overview of scores on relevant items of the SCID-IV: Module anxiety disorders

Nr.	**PANIC DISORDER**	**?**	**No**	**Ps**	**Pr**	**Yes**	**Source** [1]
F1	Recurrent unexpected panic attacks			X			230, 752, 753
F2	Persistent concern, worry implications, change in behavior			X			141, 702
F3	Not better accounted for by another disorder	X					
	**Conclusion:** Not pursued						
	**SOCIAL PHOBIA**	?	No	Ps	Pr	Yes	Source
F47	Persistent fear			X			
F53	Exposure provokes anxiety			X			
F54	Fear is recognized excessive/unreasonable	X					
F55	Feared situations are avoided			X			244
F56	Interferes with person's normal routine			X			London, 244 [2]
F57	Duration ≥6 months			X			
F58	Not substance related of medical					X	
F59	Not related to medical or other mental disorder			X			Depression [3]
	**Conclusion:** Social phobia (F47-59 yes)			X			

No = certainly not present; Ps = possibly present; Pr = probably present; Y es = certainly present; [1] The numbers refer to the letters in Van Gogh's correspondence; [2] Especially during stay in London; [3] Possibly not related to depression

Supplemental Table 5. Overview of scores on relevant items of the SCID-IV: Module substance use disorders

Nr.	**ALCOHOL USE DISORDER**	**?**	**No**	**Ps**	**Pr**	**Yes**	**Source** [1]
E1	Alcohol dependence likely					X	
	Started with abuse<br>- 1883 (The Hague)<br>- 1886 (Paris)				 X 	 <br>X	 <br>603
E2	Use results in failure to fulfill major role					X	750 (note7)
E3	Use in hazardous situations	X					
E4	Use results in legal problems					X	750 (note7)
E5	Continues use despite problems				X		738, 752, 760, 764
	E2-E4: ≥1 yes					X	
E7	More or longer use than intended				X		760
E8	Persistent desire or unsuccessful efforts to end abuse				X		
E9	Much time spent to obtain alcohol			X			
E10	Important social activities given up			X			
E11	Continues use despite problems					X	645, 694
E12	Tolerance				X		
E13	Withdrawal symptoms				X		747 (note2) (delirium)
	E7-E13: ≥3 yes within 12 months				X		
	**Conclusion:**<br>Alcohol dependence				X		

No = certainly not present; Ps = possibly present; Pr = probably present; Y es = certainly present; [1] The numbers refer to the letters in Van Gogh's correspondence

Supplemental Table 6. Conclusions from questionnaires on personality disorders

Questionnaire	Result/conclusion
Selftest 'De Viersprong'	Clear suggestions for a borderline personality disorder and/or an obsessive-compulsive personality disorder
MSI-BPD	Positive on 8 of the 10 questions; a score of 7 or higher strongly suggests borderline personality disorder
GAPD	Moderate to severe disfunction in self-pathology and severe maladaptation in interpersonal functioning, i.e. comparable to a level score of 2 and 3 respectively on the Personality Functioning Scale of the alternative DSM-5 model for personality disorder (DSM-5, part III)<br>The A-criterium is met, i.e. sufficiently high for the diagnosis/presence of a personality disorder according to the general criteria.
PID-5	Fits in a specific DSM-5, part III personality disorder, by scoring high on all criteria of a borderline personality disorder
Overall conclusion	All questionnaires provide strong indications for a personality disorder, while the combination of the different traits convincingly point into the direction of a severe borderline personality disorder. There are also traits of an obsessive-compulsive personality disorder, however not in the compulsive, controlling sense but in the sense of a perseverative, rigid perfectionism.

MSI-BPD: McLean Screening Instrument for Borderline Personality Disorder; GAPD: General Assessment of Personality Disorders; PID-5: Personality Inventory for DSM-5

Supplemental Table 7. Scores on the General Assessment of Personality Disorders (GAPD)

Domains of the GAPD	Score	Compared with normals	Compared with patients with a personality disorder
Poorly delineated interpersonal boundaries	2.45	High	Average
Lack of self clarity	2.81	High	Average
Lack of history and continuity	3.11	Very high	Average
Lack of autonomy and agency	3.92	Very high	High
Impaired capacity intimacy/attachment	3.38	Very high	High
Problems with affiliation	3.20	Very high	High
Disfunctioning of self	2.97	Very high	Average
Interpersonal dysfunction	3.29	Very high	High
Total score	3.13	Very high	High

Supplemental Table 8. Scores on the individual items of the Personality Inventory for DSM-5 (PID-5)

Items of the PID-5	Score	Compared with US normals
Emotional lability [1]	2.42	Very high
Anxiousness [1]	2.00	High
Separation insecurity [1]	2.14	High
Submissiveness	0.00	Very low
Hostility [1]	1.90	High
Perseveration	1.77	High
Depressivity [1]	1.43	High
Suspiciousness	1.17	Average
Restricted affectivity	0.57	Average
Withdrawal	1.30	Average
Intimacy avoidance	1.00	Average
Anhedonia	0.88	Average
Manipulativeness	1.00	Average
Deceitfulness	0.30	Average / low
Grandiosity	0.33	Average / low
Attention seeking	1.13	Average
Callousness	1.07	Average
Irresponsivity	1.57	High
Impulsivity [1]	1.83	High
Distractibility	0.55	Average
Risk taking [1]	2.64	Very high
Rigid perfectionism	1.10	Average
Unusual beliefs and experiences	0.13	Average / low
Eccentricity	0.77	Average
Perceptual disregulation	0.17	Average / low
**Domains of the PID-5**	**Score**	**Compared with US normals**
Negative Affectivity	2.18	Very high
Detachment	1.06	Average / high
Antagonism	0.53	Low
Disinhibition	1.32	Average / high
Psychoticism	0.36	Low

[1] Criteria of borderline personality disorder

Supplemental table 9. Neuropsychiatric history of Van Gogh

Time	Symptomatology	Letter [1]
From about 1873	- Depressive mood changes<br>- Maladjusted behavior, visits to brothels<br>- Lifestyle problems in sleeping, eating, use of alcohol and tobacco	
1878 (Amsterdam)	- Hyperreligiosity, Self-flagellation	
1879-1880 (Borinage)	- Self-neglect, active melancholy<br>- Consultation of Ramaer (neuropsychiatrist) by his parents considering admission in asylum of Geel (Belgium)<br>- Sometimes facial tics	155
1882 (The Hague)	- Nervous exhaustion, sleeplessness, headache	
1886-1888 (Paris)	- Increased abuse of alcohol<br>- Exhaustion, dazed, feels dizzy and paralyzed<br>- Nightmares<br>- Periods of uncontrolled behavior	
1888 (Arles):	- Attacks mentioned in various terms<br>- Melancholy<br>- Unaccountable, involuntary feelings<br>- Stupor on some days<br>- Sleepwalking	 628<br><br>615
December 1888-April 1890 (Arles)	- Seven (sub)acute episodes with variable combinations of symptomatology<br>o Disturbance of memory and consciousness<br>o Confusion<br>o Anxiety<br>o Exaltation<br>o Visual and auditory hallucinations<br>o Nightmares<br>o Delusions<br>o Depressions<br>o Prosopagnosia and spatial agnosia	812
1890 (Auvers)	- Suicide attempt	

1 The numbers refer to the letters in Van Gogh's correspondence

Supplemental table 10. Neuropsychiatric family history of Van Gog**h** [1]

**Family member**	**Relationship**	**Diagnosis/symptomatology**
Theo van Gogh (1857-1891)	Brother	- Dementia paralytica<br>- Two seizures in his last two days
Wilhelmina van Gogh (1862-1941)	Sister	- Schizoaffective disorder, depressive type<br>- Dementia
Cor van Gogh (1867-1900)	Brother	- Probably suicide
Clara Adriana Carbentus (1817-1866)	Mother’s sister	- Seizures from age 34 (1851)
Hendrik van Gogh (1853-1866)	Cousin	- Attacks from age 31 (1884)<br>- Dies after dropping out of a boat at age 33 (1886

[1] Voskuil P, Diagnosing Vincent van Gogh, an expedition from the sources to the present "mer à boire". Epilepsy Behav. 2013; 28:177-80.

Supplemental table 11. Arguments pro and contra epilepsy as a possible diagnosis in Van Gogh's case

**Arguments pro**		**Letter [1] /reference**
1	Minor equivalents of focal seizures with impaired awareness<br>Some examples as mentioned in Van Gogh's letters<br>- "Repeated and unexpected emotions"<br>- "Moments of anguish – above all – in my so-called mental illness"<br>- "Terrible fits of anxiety sometimes – without any apparent cause – or then again a feeling of emptiness and fatigue in the mind"	Gastaut [2]<br><br>751<br>752<br>764
2	Three 'crises' in 1889 allegedly witnessed by his guards, taken down years later by third parties	
	First' crisis': Possible focal seizure with impaired awareness evolving to bilateral tonic-clonic seizure<br>- There was a clear 'crisis' on or around July 16<br>- It started sudden and unexpected as written by Van Gogh<br>- According to Trabuc, Van Gogh had a contorted hand and looked haggard before falling on the ground<br>- Gastaut mentions no source and there is no other account like this	 <br>797<br>Trabuc as mentioned in Gastaut [2]
	Second 'crisis': Possible focal seizure with impaired awareness<br>- "An odd thing happened one day. Van Gogh and Poulet were coming back from an outing; they were just going up the stairs, when Vincent, who was in front, suddenly turned round and gave Poulet an almighty kick in the stomach. Poulet, who was used to the odd habits of the asylum inmates, said nothing about it.<br>- Next day Vincent said: "Yesterday, chief, I swung a kick at you. Please forgive me, I had the Arles police after me"	Interview of Poulet in Tralbaut [3]
	Third 'crisis': Possible focal seizure evolving to bilateral tonic-clonic seizure<br>- "Another day, when he went to fetch Vincent for dinner, Poulet found him with staring eyes and foaming lips: Vincent had been trying to eat his paints. Three tubes of them! Dr Peyron and Trabu [sic]… and Poulet only just managed to save him. This was not the only incident of the kind."	Interview of Poulet in Tralbaut [3]
3	Parts of the prolonged episodes between December 1888 and April 1890 are to some extent similar to what can be seen in limbic ictal psychosis: disturbance of consciousness, agitation, anxiety, visual and auditory hallucinations, delusions (of being persecuted or poisoned)	
4	Some characteristics of the Gastaut-Waxman-Geschwind syndrome prominent in the personality of Van Gogh: hyperreligiosity, hypergraphia and tendency to elation	
5	Family history of epilepsy	
**Arguments contra**		
1	The testimonies mentioned under Pro 2. can be questioned<br>- Gastaut does not mention a source for Trabuc's account<br>- Correspondence between Voskuil and Gastaut (1988) reveals that Gastaut had lost his documentation<br>- Tralbaut interviewed Poulet when he was 90 years old.	
2	There are no indications that other focal impaired awareness or bilateral tonic-clonic seizures ever have taken place	
3	The duration of an un-interrupted period of symptoms within the seven prolonged intermitting episodes is not known	
4	In the family history interpretation from sources is, with some exceptions, ambiguous	

[2.] Gastaut H. La maladie de Vincent van Gogh envisagée de la lumière des conceptions nouvelles sur l'épilepsie psychomotrice [Vincent van Gogh's disease seen in the light of new concepts of psychomotor epilepsy]. Ann Med Psychol (Paris). 1956; 2: 1-43.

[3] Tralbaut ME. Vincent van Gogh. Edita Lausanne; 1974: 288-90.
